# Efficacy of Single Titanium Elastic Nail in the Treatment of Child Femur Fractures

**DOI:** 10.7759/cureus.46099

**Published:** 2023-09-27

**Authors:** Cafer Ozgur Hancerli, Haluk Agus

**Affiliations:** 1 Orthopedics, Kanuni Sultan Süleyman Training and Research Hospital, Istanbul, TUR; 2 Orthopedics, Tepecik Training and Research Hospital, Izmir, TUR

**Keywords:** fracture in a child, union, fracture, elastic nail, femur

## Abstract

Background: Femoral shaft fractures in childhood constitute an important part of emergency visits to orthopedic clinics. The aim of this study was to investigate the efficacy and reliability of a method in the treatment of simple fractures in the middle of the femoral diaphysis in patients with multiple injuries or in the treatment of cases with a medullary canal that is too narrow for two nails, with a single stretch nail thicker than half the diameter of the canal.

Methods: Between July 2002 and November 2006, examinations were made of 11 femoral fractures in 11 patients who were admitted to the pediatric emergency department of Tepecik Training and Research Hospital with a diagnosis of femoral fracture and who were hospitalized and treated with a single flexible intramedullary titanium nail.

Results: In the follow-up, no problem was found in terms of union and length difference in any of the cases, except for a 10º varus deformity present after surgery in one case.

Conclusions: Elastic intramedullary nailing is an effective method in the treatment of simple femoral fractures in children. A single elastic nail provides adequate stability following open reduction with minimal incision in patients whose short operating time must be kept short such as those with head trauma, thoracic trauma, or intra-abdominal pathology, or patients with a narrow medullary canal where two flexible nails cannot pass. We think that it is a feasible method because it causes minimal soft tissue damage.

## Introduction

The placement of two titanium elastic nails (TENs) applied with two incisions over the distal metaphysis has become the traditional method of treatment in femoral diaphyseal fractures in patients between the ages of 5 and 11. With the increase in the use of TENs, advantages such as shorter hospital stays, reduced risk of re-fractures, early mobilization, and less blood loss have been achieved [1,2].

In order to increase stability, methods have also been described with a third TEN applied from the lateral or medial side, or with four TENs (two medial and two lateral) applied from the lateral and medial sides, and, citing cosmetic problems, different methods have been proposed such as two TENs applied with a single incision from the lateral side instead of two separate incisions from the medial and lateral sides [3,4].

There are no studies of femoral fractures treated with a single TEN. The reason for this may be that a single TEN may lead to complications such as angulation, shortening, and rotation defects, especially in unstable fractures, and early weight-bearing failure.

In this study, an examination was made of the results in terms of the efficacy and safety of the method of treatment with a single TEN wider than half the diameter of the canal in patients with simple fractures in the middle of the femoral diaphysis accompanied by multiple injuries, or in cases with a medullary canal which was too narrow for two nails, or to shorten the duration of the operation.

This article was previously presented as a meeting abstract at the Hippocrates Congress on Medical and Health Sciences on March 4-5, 2022.

## Materials and methods

Material and method

Our article is a retrospective study. Pediatric patients with femur fractures who were treated by a single TEN at Tepecik Training and Research Hospital between July 2002 and November 2006 were evaluated retrospectively. Patients with pathological fractures or neuromuscular disorders and patients who were not followed up for bone union were excluded from the study.

A single TEN was used in order to shorten the operation time in seven patients with additional injuries, and in the remaining four patients because the medullary canal was too narrow to insert two nails (Table 1).

**Table 1:** Intramedullary diameter, TEN diameter, and additional Injury TEN: titanium elastic nail

	Age	Side	Intramedullary diameter	TEN diameter	Mechanism of injury	Additional injury	Reason for using single TEN
Case 1	7	Left	9 mm	4.5 mm	Falling from a height	Head trauma	Having additional injuries
Case 2	4	Right	5.7 mm	2.5 mm	Other		Narrow intramedullary canal
Case 3	7	Right	8 mm	4 mm	Traffic accident	Cervical spine fracture	Having additional injuries
Case 4	9	Left	9 mm	4 mm	Falling from a height	Head trauma	Having additional injuries
Case 5	11	Left	7.2 mm	4 mm	Traffic accident		Narrow intramedullary canal
Case 6	9	Right	7.8 mm	3.5 mm	Traffic accident	Radius break and narrow intramedullary canal	Narrow intramedullary canal
Case 7	11	Right	8 mm	3.5 mm	Traffic accident	Tibia fracture	Having additional injuries
Case 8	7	Right	8.9 mm	4.5 mm	Traffic accident	Head trauma	Having additional injuries
Case 9	7	Right	9 mm	4 mm	Falling from a height	Clavicle fracture and thoracic trauma	Having additional injuries
Case 10	6	Left	5.2 mm	3 mm	Traffic accident		Narrow intramedullary canal
Case 11	8	Left	9 mm	4 mm	Traffic accident	Clavicle fracture and thoracic trauma	Having additional injuries

Surgical technique

In the selection of the nails, care was taken to ensure that the diameter of the nail was greater than half that of the femoral canal (Table 1). Medullary canal diameter was measured by direct radiography and the elastic nail was shaped by bending to be half the diameter and three times the width of the medullary canal. Fracture reduction was performed with a mini-open incision. The elastic nail was advanced around the greater trochanter to the cortical bone. During the operation, fracture reduction was evaluated by fluoroscopy, and the length was compared with the healthy side. At the end of the operation, a plaster splint with pelvic support was applied with the ankle in a neutral position, the knee at 30º of flexion, and the hip at approximately 15º of flexion.

Post-operative treatment

In the postoperative period, direct radiographs of the patients were taken without removing the splints. Angulations in the anterior, posterior, and lateral planes and overlapping of broken parts were measured on the radiographs. On the second postoperative day, femoral anteversion angles of the healthy and broken sides were measured by computed tomography (CT). Patients in good general condition were mobilized with double crutches without putting any weight on the broken side. Radiographs were checked on the fifteenth, thirtieth, and forty-fifth days, and at the third, sixth, ninth, and twelfth months, and evaluated clinically.

The splints of patients with callus formation in two or three cortexes in re-examination were removed and passive movement was initiated in the ankle, knee, and hip joints. When the full union was seen, load was applied. At the sixth month follow-up of the patients, range of motion was evaluated. In the sixth month, femoral anteversions of the healthy and broken sides were measured with CT guidance and compared with the initial values.

At the final check (two years after surgery), the range of motion and walking of the patients were observed clinically and their femoral anteversions were measured clinically. Femoral anteversion measurement was made in the prone position, with hip extension and the knee flexed 90 degrees; the hip was forced into internal rotation, and the angle between the tibia and the vertical plane was measured when the trochanter major was most prominent (Craig method). The femoral length difference was evaluated by measuring the length between the spina iliaca anterior superior and the medial malleolus.

## Results

Eight of our patients were boys and three were girls. Their average age was 7.8 years. All patients had simple fractures. Seven of our patients had been injured in traffic accidents, three by falling from a height, and one was a compression injury (Table 2).

**Table 2:** Demographic data

	Patients (n:11)
Gender	Male-8
	Female-3
Fracture side	Right-6
	Left-5
Age	7.8 (4-11)
Injury type	Traffic accident-7
	Falling from a height-3
	Other-1
Fracture type	32A1-2
	32A2-8
	32A3-1
Fracture union (days)	56 (30-120)
Follow-up time (months)	73

Additional injuries were present in seven patients (64%); ipsilateral tibia fracture in one patient, ipsilateral clavicle fracture in two patients, transverse process fracture of the sixth cervical vertebra in one patient, radius diaphyseal fracture in one patient and head trauma in two patients. One of these was operated on by neurosurgeons and transferred 10 days after the injury. The average operation time was 72 (35-100) minutes. The patients were operated on when their general condition was appropriate, after an average of five days (1-15 days).

Postoperative direct radiographs showed an angulation of 0-10 (4.4 ± 4.1) degrees and 0-7 mm of overlap on anterior-posterior radiography, and an angulation of 0-8 (1.2 ± 2.8) degrees on the lateral radiograph (Table 3).

**Table 3:** Angulations and length differences

	Anteroposterior radiography	Lateral radiography	Overlap
Case 1	3°	0°	
Case 2	0°	0°	5 mm
Case 3	8°	0°	
Case 4	0°	0°	
Case 5	0°	0°	
Case 6	10°	6°	7 mm
Case 7	4°	0°	
Case 8	7°	0°	
Case 9	10°	8°	
Case 10	0°	0°	3 mm
Case 11	7°	0°	

There was no significant difference between the femoral anteversion grade measured on the second day and on the sixth month after surgery. The mean anteversion angles measured on the second postoperative day were 18° (1° - 35°) and the mean anteversion angles measured in the sixth month were 16° (1° - 32°) (p = 0.34) (Table 4).

**Table 4:** Femoral anteversion measurements

	Anteversion second day	Anteversion sixth month
Case 1	28°	32°
Case 2	18°	15°
Case 3	4°	4°
Case 4	30°	24°
Case 5	23°	18°
Case 6	5°	10°
Case 7	1°	1°
Case 8	20°	10°
Case 9	12°	21°
Case 10	35°	32°
Case 11	21°	7°
Standard deviation	1°-35° 17.9±11.2	1°-32° 15.8±10.6

At the final checkup, the hip and knee joint movements of the patients were complete and painless, and there was no problem with walking. Clinically measured femoral anteversions were evaluated as normal. Five patients had an elongation in the femoral length, one patient had a shortened femoral length, and five patients had no change. The elongation in femoral length was 5.5 mm (7 mm shortening - 20 mm elongation) (Table 5).

**Table 5:** Clinical measurements at 12 months

	Broken side	Right femur anteversion	Left femur anteversion	Length difference
Case 1	Left	32°	38°	Left 0.5 cm long
Case 2	Right	37°	35°	No shortness
Case 3	Right	32°	35°	No shortness
Case 4	Left	40°	43°	No shortness
Case 5	Left	40°	40°	Left 2 cm long
Case 6	Right	50°	40°	Right 1 cm long
Case 7	Right	38°	32°	No shortness
Case 8	Right	62°	45°	Right 2 cm long
Case 9	Right	62°	44°	No shortness
Case 10	Left	45°	40°	Left 1 cm short
Case 11	Left	36°	34°	No shortness

The mean time to union was 56 days (Table 6). Radiologically, 10º varus angulation continued in only one patient (case 6) on anterior-posterior radiography. There was no angulation in the anterior and lateral radiographs of the other patients (Table 7).

**Table 6:** Union time

	Union time (days)
Case 1	60
Case 2	31
Case 3	36
Case 4	57
Case 5	120
Case 6	83
Case 7	51
Case 8	30
Case 9	49
Case 10	43
Case 11	60
Average	56

**Table 7:** Remodeling

	Remodeling
Case 2	280 days
Case 3	730 days
Case 6	10° varus
Case 8	140 days
Case 9	280 days
Case 11	348 days

## Discussion

It was concluded that in children aged 4-11 with simple femur diaphysis fractures, treatment can be performed with a single TEN in order to shorten the operation especially in cases with accompanying multiple injuries, or in cases in which the medullary canal is too narrow for the usage of two TENs.

The American Academy of Orthopedic Surgeons (AAOS) clinical practice guide has accepted TEN as a feasible treatment with minimal complications in pediatric femur fractures [1,2,5-9]. When two TENs are applied, they should fill 80% of the femoral canal diameter [8,10]. A TEN which is applied by twisting into a double C-type configuration is a biomechanically superior fixation method for long bone diaphysis fractures [11]. In our study, a TEN was applied so as to fill more than half of the femoral canal diameter and was inserted into the apophysis in the greater trochanter to increase stability. The greater trochanter apophysis does not contribute to longitudinal growth. For this reason, it has been reported that removing one TEN temporarily from the greater trochanter will not cause serious consequences [12].

The biggest problem that can be encountered in a single TEN application is the lack of rotational and angular stability. In our study, a TEN wider than half the diameter of the canal was used in order to increase stability, and stabilization was increased by inserting the TEN proximal to the greater trochanter apophysis. In the postoperative period, keeping the extremity in a long leg plaster splint until sufficient callus tissue was seen also contributed to the stability.

The main advantages of TEN application are early mobilization, shorter hospital stay, reduced risk of repeat fractures, less blood loss, and shortened operation time [13-16] Although the operation times differ in other studies, 0.9 hours [14] have been reported for TEN, 1.4-1.9 hours [17-19] for submuscular plating, and 1.9 hours [18] for rigid plating. In our study, the average operation time was 1.2 hours.

Cage et al. compared patients aged 6-11 with femoral fractures, to whom two elastic nails were applied laterally, with patients to whom one nail was applied laterally and one medially [20]. They maintained that while there was no difference between the clinical results of the two methods, the operation time was shortened by 30 minutes [20]. Knedel et al. did not observe any difference between these two methods in terms of operating time [4].

Cha et al. reported adequate stability and recovery in a study in which they inserted TENs into the epiphysis of the greater trochanter or femoral neck cortex in unstable subtrochanteric femur fractures [12].

The strength of this study is that we followed up with all our patients as closely as possible and for as long as possible. A limitation of this study is that patients' weights were not recorded.

## Conclusions

In conclusion, it is known that two elastic intramedullary nailings are an effective method in the treatment of simple femur fractures in children. In our study, it was revealed that rotational stability was achieved with a single elastic intramedullary nailing and long leg splint. We believe that treatment with a single titanium flexible nail can be applied when necessary to children between the ages of 4 and 11. We think that the correctness of our thesis will increase if the number of patients is increased and larger series are presented in new studies.
